# Different Impacts of Cardiovascular Risk Factors on Oxidative Stress

**DOI:** 10.3390/ijms12096146

**Published:** 2011-09-20

**Authors:** Maria L. Mansego, Josep Redon, Sergio Martinez-Hervas, Jose T. Real, Fernando Martinez, Sebastian Blesa, Veronica Gonzalez-Albert, Guillermo T. Saez, Rafael Carmena, Felipe J. Chaves

**Affiliations:** 1 Genotyping and Genetic Diagnosis Unit, Research Foundation of Hospital Clínico; Avenida Blasco Ibañez, 17, Valencia 46010, Spain; E-Mails: Sebastian.Blesa@uv.es (S.B); veronica.gonzalez@uv.es (V.G.-A.); felipe.chaves@uv.es (F.J.C.); 2 CIBER of obesity (CIBERob), Santiago de Compostela 15706, Spain; E-Mails: Josep.redon@uv.es (J.R.); fernandoctor@hotmail.com (F.M.); 3 Hypertension Unit, Hospital Clinico; Avenida Blasco Ibañez, 17, Valencia 46010, Spain; 4 Service of Endocrinology and Nutrition, Hospital Clínico Universitario, Avenida Blasco Ibañez, 17, Valencia 46010, Spain; E-Mails: Sergio.Martinez@uv.es (S.M.-H.); Jose.T.Real@uv.es (J.T.R.); Rafael.Carmena@uv.es (R.C.); 5 CIBER de Diabetes y Enfermedades Metabólicas Asociadas (CIBERDEM), Barcelona 08017, Spain; 6 Department of Biochemistry and Molecular Biology, University of Valencia, Avenida Blasco Ibañez, 17, Valencia 46010, Spain; E-Mail: Guillermo.Saez@uv.es

**Keywords:** oxidative stress, glutathione peroxidase, superoxide dismutases, mRNA, hypertension, familial hypercholesterolemia, combined familial dyslipidemia

## Abstract

The objective of the study was to evaluate oxidative stress (OS) status in subjects with different cardiovascular risk factors. With this in mind, we have studied three models of high cardiovascular risk: hypertension (HT) with and without metabolic syndrome, familial hypercholesterolemia (FH) and familial combined hyperlipidemia (FCH) with and without insulin resistance. Oxidative stress markers (oxidized/reduced glutathione ratio, 8-oxo-deoxyguanosine and malondialdehide) together with the activity of antioxidant enzyme triad (superoxide dismutase, catalase, glutathione peroxidase) and activation of both pro-oxidant enzyme (NAPDH oxidase components) and AGTR1 genes, as well as antioxidant enzyme genes (CuZn-SOD, CAT, GPX1, GSR, GSS and TXN) were measured in mononuclear cells of controls (*n* = 20) and patients (*n* = 90) by assessing mRNA levels. Activity of some of these antioxidant enzymes was also tested. An increase in OS and pro-oxidant gene mRNA values was observed in patients compared to controls. The hypertensive group showed not only the highest OS values, but also the highest pro-oxidant activation compared to those observed in the other groups. In addition, in HT a significantly reduced antioxidant activity and mRNA induction of antioxidant genes were found when compared to controls and the other groups. In FH and FCH, the activation of pro-oxidant enzymes was also higher and antioxidant ones lower than in the control group, although it did not reach the values obtained in hypertensives. The thioredoxin system was more activated in patients as compared to controls, and the highest levels were in hypertensives. The increased oxidative status in the presence of cardiovascular risk factors is a consequence of both the activation of pro-oxidant mechanisms and the reduction of the antioxidant ones. The altered response of the main cytoplasmic antioxidant systems largely contributes to OS despite the apparent attempt of the thioredoxin system to control it.

## 1. Introduction

An excessive production of reactive oxygen species (ROS) outstripping antioxidant defense mechanisms has been implicated in conditions which impact the cardiovascular system and the development of atherosclerosis [1,2]. An excess of ROS in the blood system as well as in several other cellular systems [3], including vascular wall cells [4] and circulating blood cells [5], has been described in subjects with advanced atherosclerosis. The increased oxidative stress (OS) is driven by the presence of the so-called cardiovascular (CV) risk factors and their impact on both pro-oxidant and antioxidant mechanisms. The CV risk factors, hypertension (HT), familial hypercholesterolemia (FH) and familial combined dyslipidemia (FHC), which produce and accelerate atherosclerosis also have increased OS levels [6–8]. The individual impact on the underlying OS mechanism of each CV risk factor, however, is not well known.

In a previous study carried out in hypertensive patients by our group, we observed that both blood and peripheral mononuclear cells exhibit important deficiencies of physiological antioxidants with a deep reduction in enzymatic activity and GSH levels [9]. Mechanisms underlying these alterations are not well understood, but an increase in activity of pro-oxidant enzymes, mainly NADPH oxidase that can be activated by angiotensin II through Angiotensin AT1 receptor (AGTR1) stimulation, has been implicated in the high level of ROS in several cellular models of hypertensive subjects [10,11]. Likewise, an inadequate response of the main cytoplasmic antioxidant systems has been described in studies reported by our group [7]. An increment in pro-oxidant enzyme activity increases ROS production, it may saturate the capacity of antioxidant enzymes and leads to high generation of OS [12,13].

Whether or not the hyperactivity of the pro-oxidant and a reduction of the antioxidant mechanisms observed in hypertensives also exist in the presence of other diseases and CV risk factors has not been previously addressed. Thus, the objectives of the present study were to assess OS status, cytoplasmic antioxidant enzymatic activity and the activation of pro-oxidant and antioxidant genes in subjects with different CV risk factors: HT, FH (model of pure hypercholesterolemia) and FCH (model of mixed dyslipidemia) avoiding their overlapping.

## 2. Results and Discussion

### 2.1. General Characteristics of the Study Population

The study was performed in 90 patients with CV risk factors (43 HT, 17 FH and 30 FCH) and 20 control volunteers. Twenty-two of the hypertensives had metabolic syndrome (MS) and 17 of the FCH had insulin resistance (IR). The characteristics of each group of patients and controls are shown in Table 1 and in Table S2 for patients grouped by MS or IR. Controls were normotensives and had slightly lower total-cholesterol levels than hypertensives. In addition, due to selection criteria, the controls as well as those with HT had significantly lower levels of total cholesterol or triglycerides than did those with FH or with FCH.

### 2.2. Oxidative Stress and Antioxidant Enzyme Activity

OS parameters and the antioxidant enzyme activity in the study groups and controls are shown in Table 2. Mononuclear cells from HT subjects showed the lowest GSH and the highest GSSG values among the control, FH and FCH groups, after adjustment for age, gender and BMI. Likewise, 8-oxo-dG, a byproduct of ROS-induced DNA damage, was also significantly increased in hypertensive subjects as compared to the other groups. The OS degree of FH and FCH, even though it was significantly higher than that observed in controls, was lower than that observed in HT. Besides the increment in the oxidative status, there was a significantly lower activity level of the cytoplasmic antioxidant enzymes *SOD*, *GPx1* and *CAT* in HT when compared to that observed in controls and in the other two patient groups (Table 2). The reduced activity observed in HT was also present in FH and FCH, although the extent of the reduction was significantly lower than that observed in HT. In fact, only the activity of GPx1 and CAT was significantly lower in HT than it was in controls. The presence of MS in HT group did not increase OS or reduce the antioxidant enzyme activity. However, the values of GSSG and GSSG/GSH ratio were higher in the subgroup of IR than non-IR subjects in the FCH (Table S2).

### 2.3. mRNA Levels of Pro-Oxidant Genes

**Figure 1:**
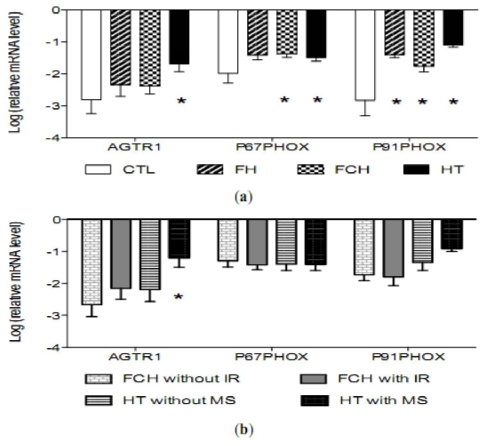
Angiotensin AT1 receptor (AGTR1) and some components of the NADPH oxidase (P91PHOX, P67PHOX) log ratio relative mRNA values in mononuclear cells of (**a**) controls (*n* = 20, CTL), familial hypercholesterolemia (*n* = 17, FH), familial combined hyperlipidemia (*n* = 30, FCH) and hypertensives (*n* = 43, HT); and (**b**) FCH without insulin resistance (*n* = 13, FCH without IR), FCH with insulin resistance (*n* = 17, FCH with IR), HT without metabolic syndrome (*n* = 21, HT without MS) and HT with metabolic syndrome (*n* = 22, HT with MS) of the study population. * *p* values denote differences between controls and disease. Statistical tests: Multivariate linear regression analyses adjusted by age, gender and BMI. NOTE: A gene is up-regulated when their relative values are higher in the disease group than controls. However, if the values are lower ones, the gene is down-regulated.

The mRNA levels of AGTR1 gene and of P22PHOX, P91PHOX, P47PHOX, P67PHOX and RAC1 genes as components of the NADPH oxidase was analyzed in the mononuclear cells and adjusted for age, gender and BMI. As shown in Figure 1a, AGTR1, *P*67*PHOX* and *P91PHOX* mRNA levels were significantly higher in HT compared to controls and FCH were higher to controls in *P*67*PHOX* and *P91PHOX* mRNA levels. Furthermore, HT with metabolic syndrome displayed the highest values of AGTR1 mRNA, Figure 1b. No differences between patients and controls were observed for *P22PHOX*, *P47PHOX and RAC1* mRNA levels after adjusting for age, gender and BMI.

### 2.4. mRNA Levels of Antioxidant Enzymes

**Figure 2:**
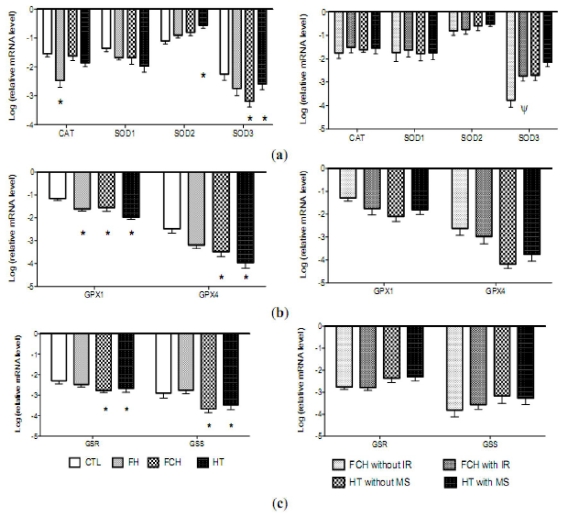
Log ratio relative mRNA values for (**a**) catalase (CAT), intracellular (SOD1), mitochondrial (SOD2) and extracellular (SOD3) copper zinc superoxide dismutase; (**b**) glutathione peroxidase system (GPX1, GPX4); and (**c**) some components of the glutathione regeneration (GSR, GSS) in mononuclear cells of controls (*n* = 20, CTL), familial hypercholesterolemia (*n* = 17, FH), familial combined hyperlipidemia (*n* = 30, FCH) and hypertensives (*n* = 43, HT) and FCH without insulin resistance (*n* = 13, FCH without IR), FCH with insulin resistance (*n* = 17, FCH with IR), HT without metabolic syndrome (*n* = 21, HT without MS) and HT with metabolic syndrome (*n* = 22, HT with MS) of the study population. * *p* values denote differences between controls and disease. Ψ *p* values denote differences between Non-IR and IR. Statistical tests: Multivariate linear regression analyses adjusted by age, gender and BMI. NOTE: A gene is up-regulated when their relative values are higher in the disease group than controls. However, if the values are lower ones, the gene is down-regulated.

**Figure 3:**
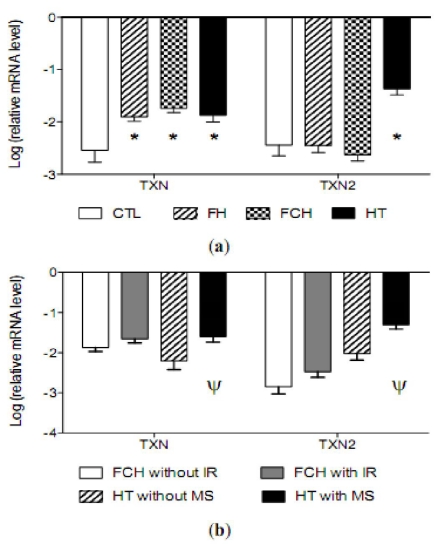
Thioredoxin (TXN) and mitochondrial thioredoxin (TXN2) log ratio relative mRNA values in mononuclear cells of (**a**) controls (*n* = 20, CTL), familial hypercholesterolemia (*n* = 17, FH), familial combined hyperlipidemia (*n* = 30, FCH) and hypertensives (*n* = 43, HT); and (**b**) FCH without insulin resistance (*n* = 13, FCH without IR), FCH with insulin resistance (*n* = 17, FCH with IR), HT without metabolic syndrome (*n* = 21, HT without MS) and HT with metabolic syndrome (*n* = 22, HT with MS) of the study population. Values are mean (SE). * *p* values denote differences between controls and disease. Ψ *p* values denote differences between Non-MS and MS. Statistical tests: Multivariate linear regression analyses adjusted by age, gender and BMI. NOTE: “without” can be abbreviated as “w/o”–HT w/o MS). A gene is up-regulated when their relative values are higher in the disease group than controls. However, if the values are lower ones, the gene is down-regulated.

The mRNA levels of the antioxidant enzymes CAT, GPx1, glutathione peroxidase 4 (phospholipid hydroperoxidase) (GPx4), intracellular (SOD1), mitochondrial (SOD2) and extracellular (SOD3) Cu-Zn superoxide dismutase and two key enzymes in the synthesis and regeneration of glutathione, glutathione synthase (GSS) and glutathione reductase (GSR), are shown in Figure 2. The mRNA levels of SOD3, GPX1, GPX4, GSS and GSR mRNAs were significantly lower in patients from the FCH and HT groups compared to controls. In patients with FH, only the levels of GPx1 were significantly lower than those observed in controls. The presence of metabolic syndrome or insulin resistance did not change the results found in the HT or FCH groups except in SOD3, Figure 2. Furthermore, TXN mRNA levels were significantly higher in all groups of patients compared to those for controls, while high values of SOD2 and TXN2 were observed only in the HT group, Figures 2 and 3. Finally, an increase in TXN and TXN2 mRNA levels were found in HT patients with metabolic syndrome, while no differences were found in the FCH subgroups (Figure 3b).

### 2.5. Discussion

The present study was designed to simultaneously assess the OS levels and the mRNA expression of the main antioxidant enzymes and their enzymatic activity in three groups of CV risk patients, HT, FH and FCH, in the presence of additional risk factor as MS and IR. A limitation of the study is that the HT and FCH groups overlap in the levels of glucose, an important risk factor, but the blood pressure is the differential factor between groups. In addition, we have observed that FCH patients present higher levels of total cholesterol and triglycerides parameters involved in the modulation of the OS. Similarly, the activation of some of the pro-oxidant enzymes was also measured. The three CV risk conditions had an increase in OS, although the highest level corresponded to the HT patients. The data from the present study were in agreement with previous reports in which atherosclerosis and cardiovascular diseases were associated with reduced GSH [14] and elevated 8-oxo-dG [15,16]. In addition to the increased OS, antioxidant activity of cytoplasmic enzymes was reduced. The highest OS observed in HT was driven by the highest activation of pro-oxidant enzymes and the lowest activity of anti-oxidant enzymes. Furthermore, OS is presently accepted as a likely causative factor in the development of insulin resistance [17,18]. This is due to prolonged exposure to ROS affects transcription of insulin receptor substrate-1 by involving serine/threonine phosphorylation. These data are in agreement with the observed increased OS in patients with IR versus non-IR from our study and in Martinez-Herbas *et al.* [6]. Moreover, we have not observed an increase in OS between hypertensive subjects with and without MS. However, mRNA levels of the TXN system increased in MS. It is known that the main pathogenic mechanism of metabolic syndrome relies on insulin resistance, low-level inflammation, and oxidative stress [19] although other factors may influence such as obesity. In fact, this is a limitation of our study because the HTA–Non-MS group has a higher percentage of obesity greater than the MS subgroup. Consequently, we used a no appropriate control subcohort to separate out the contribution of HTA and MS to oxidative stress markers in mononuclear cells.

The increased OS level was associated with an increment in some of the pro-oxidant mechanisms, NADPH oxidase, and to a reduced antioxidant enzyme activity in the cytoplasm. The low enzymatic activity of *SOD, GPx1 and CAT* can be explained partly as a consequence of the modulation of OS on antioxidant enzymes and/or an inadequate response. The evidence favoring the existence of an inadequate response was supported by the low mRNA levels of the enzymes despite the increased OS.

The present study was performed in three groups of patients in which the selection criteria allowed us to separately study the impact of each risk factor on the OS, avoiding the overlapping of these conditions, mainly between HT and FCH. A large number of methods have been used to assess oxidative stress in biological systems. The methods used have analyzed the bioavailability of the most important antioxidant mechanisms including not only the GSH and GSSG amount, but also the enzymatic activity of SOD, CAT and GPx1 together with the OS byproduct 8-oxo-dG and MDA. The OS parameters used in these studies were selected based on their recognized value and reproducibility [20–23]. We acknowledge that measured OS corresponds to the circulating cells which do not necessarily reflect what occurs in the vascular wall, although they also impact the vascular wall.

Antioxidant enzymes constitute and represent an important part of the total antioxidant activity of aerobic cells. The coordination of their functions results in the maintenance of ROS below critical levels of incompatibility with cell viability and performance. Every one of these enzymes is susceptible to ROS inactivation, and their oxidation leads to a high ratio of degradation of the enzymes by proteosome [24–27]. Furthermore, either directly or through derived products, ROS regulates the expression of antioxidant genes as a part of an adaptive response [28,29]. In this sense, hydrogen peroxide produces changes in SOD3 and SOD1 activity and fatty acid levels can modulate catalase activity [30–36].

The mechanisms operating in the OS increment for the three groups of patients showed a similar pattern even though their intensities differ. The reduction of antioxidant enzyme activities may result from the inactivation of enzymes by increased ROS, an inhibitory mechanism of gene expression conditioned by the pathologic milieu or both.

Up to now, the increased OS has been attributed to the overactivity of mechanisms that increase ROS production. Enhanced ROS-induced oxidative stress, which is mainly mediated by superoxide and hydroxyl radicals, occurs in a wide variety of human and animal hypertension models [37–39], HF [40,41] and FCH [6,16,42]. Superoxide, one of the most active ROS in the vascular wall, enhances the activity of myeloperoxidase and may activate xanthine oxidase [43]. Furthermore, this radical is produced mainly by the hyperactivity of *NADPH oxidase*, a pro-oxidant enzyme that can be enhanced by angiotensin II through the activation of AGTR1. Overactivity of angiotensin II has been described not only in hypertension but also in the presence of elevated levels of cholesterol. Indeed, cholesterol levels were positively related to the AGTR1 expression in smooth muscle cells. According to this hypothesis, we found that in mononuclear cells the increment in OS was accompanied by an increase in the expression of AGTR1 and some key components of *NADPH oxidase*, P91PHOX and P67PHOX. The activation of pro-oxidant mechanisms, resulting in ROS overproduction, can reduce the activity of the anti-oxidant enzymes as a consequence of enzyme inactivation and induced degradation by its own byproducts [44] or other free radicals [45].

An alternative explanation for the high OS levels observed, which does not exclude the above, is the existence of primary impairment of antioxidant enzymatic activity. The low mRNA levels observed, which support this alternative explanation, can only be the result of an impaired expression response since no important post-transcriptional or post-translational regulation of these enzymes has been described. This impaired expression may result from abnormalities linked to the disease state or may be secondary to chronic OS itself. It is well established that ROS regulates the expression of antioxidant and many genes as a part of an adaptive response [28,29], although the possibility of inducing the suppression of gene response also exists [46,47]. Our previous results showed an abnormality in the impact of antihypertensive treatment in OS, enzymatic activity, mRNA values and the implication of the xanthine oxidase gene polymorphisms to OS levels and blood pressure [7,48]. In a previous study of our group, the low mRNA values were maintained even when the ROS levels decrease and the bioavailability of the enzymes was at normal levels. At similar OS levels, mRNA and protein *SOD* were significantly lower in treated hypertensives than in controls [7]. Moreover, the downregulation of *SOD* has been described in other conditions with increased OS, such as in the kidney and liver of rats with chronic renal failure induced by renal mass reduction [49].

A simultaneous increment in ROS production and the impairment of the response in the main antioxidant systems contribute to an increased level of OS with consequences for lipids, proteins and DNA. Whether or not treatment for these conditions can reduce OS and normalize the mechanisms involved is a matter of debate. In our previous studies, antihypertensive treatment reduced OS, a reduction that was greater when treatment was maintained during one year, but the downregulation of the mRNA levels was still present. Moreover, the reduction of cholesterol levels lowers OS and attenuates pro-atherogenic signaling pathways in a mouse model [50]. Furthermore, the treatment with statins and fenofibrate, apart from the improvement in the lipid profile, may increase GPX antioxidant capacity [51]. It decreases concentrations of OS and inflammatory markers on diabetes-related microvascular diseases [52], increases bioavailability of nitric oxide in atherosclerotic arterial walls and activates nitric oxide synthase [53]. In addition, some studies have linked ROS production and OS to insulin resistance [54–56]. Through *in vitro* studies and in animal models of diabetes, it has been found that antioxidants improve insulin sensitivity [57,58].

In contrast to the abnormalities observed in the main antioxidant mechanisms, the response of the thioredoxin system seems to be normal, with an increment in the mRNA levels. In contrast to the GSH system, mRNA levels of the TXN and SOD2 were significantly higher in the hypertensive group than in controls. Thioredoxin is a small protein with two isoforms encoded by two different genes, TXN and TXN2. Regulation of the GSH and TXN systems seems to be independent, but interaction between the two has been described [59]. The disparity observed between the two thiol-systems could be explained by the different kind of regulation for each of the systems. The protection mechanism during periods of increased oxidative stress, activating the TXN system, would be the presence of such ROS as peroxynitrite. It might induce thioredoxin reductase expression in endothelial cells, possibly as a protective mechanism during oxidative stress [60]. Furthermore, it is important to note the induction in the mRNA levels of SOD2. This enzyme is known to be activated through thioredoxins [61] and have an important role played by the cytokine inducible enhancer locus in the Mn-SOD gene. Moreover, Mn-SOD is highly regulated at post-translational levels [62].

## 3. Experimental Section

### 3.1. Selection of Study Participants

Patients with HT, FH and FCH were invited to participate if the following criteria were met: (a) for hypertension, essential hypertension was defined according to the criteria of the VII Joint National Committee [63] and as previously reported [15]; (b) for familial hypercholesterolemia, criteria included: plasma levels of total and LDL-C above the 95th percentile corrected for age and sex, presence of tendon xanthomata, coronary heart disease in the index patient or in a first-degree relative and bimodal distribution of total and LDL-C levels in the family indicating an autosomal dominant pattern of phenotype IIa [64]; (c) for FCH, the diagnosis was based on the presence of hyperlipidemia (cholesterol and triglyceride concentration above the 90th percentile for our population corrected for age and gender) and plasma apo B (>1.20 g/L) in the index patient, together with variable phenotypes IIa, IIb or IV in the first degree relatives, a family history of arteriosclerosis and absence of xanthomas in the patient and in first degree family members [65]. A group of healthy, normotensive, non-smokers were selected as a control group.

Allocation to one group implies the absence of criteria for pertaining to the other groups, and none of the patients were ever treated for the underlying disease or the medication was withdrawn at least six weeks before the study, provided that no risk to their health. Subjects were under medical supervision throughout the study.

In addition, HT subjects were subgrouped by the presence or the absence of MS [63]. FCH subjects were subgrouped into groups of those with or without IR, as defined by the HOMA index, of higher than the 75th percentile of the homeostasis model assessment index (P75th HOMA = 3.2) of our population [66]. Those patients with diabetes mellitus were excluded. The Ethics Committee of the Hospital approved the study, and the participants gave their informed consent.

Blood pressure was measured using a mercury sphygmomanometer in the office according to the recommendations of the British Hypertension Society [67], and using an oscillometric monitor (Spacelabs 90202 or 90207) in ambulatory conditions over 24 h on a regular working day. Blood samples were obtained in the morning after a minimum of 8 h fasting. Serum biochemical profiles were measured using an autoanalyzer.

### 3.2. Analytical Procedures

Markers of oxidative stress were determined in circulating mononuclear cells isolated by Ficoll-Hypaque methods as previously reported [68]. Oxidized and reduced glutathione (GSSG and GSH) were analyzed by high performance liquid chromatography (HPLC) and GSH was analyzed by the glutathione-S-transferase assay [69]. In addition, we calculated as the mean of the GSSG:GSH ratios determined in individual subjects. Malondialdehide was analyzed by HPLC and spectrophotometric quantification of the MDA-TBA at 532 nm [70]. The protein content was measured using the Bradford method. DNA damage, assessed by the formation of 8-oxo-7,8-dihydro-2-deoxyguanosine (8-oxo-dG), was quantified by HPLC-EC detection after its complete enzymatic digestion [71]. Total superoxide dismutase activity was determined by spectrophotometry [72] as well as catalase [73] and glutathione peroxidase 1 (GPx1) [74] activities.

### 3.3. DNA Extraction and 8-oxo-Deoxyguanosine Measurement

Cell DNA was isolated by means of the Gupta method, with the modification described by Muñiz, [71]. In which chloroform isoamyl alcohol (24:1) is used instead of phenol for the removal of proteins. Isolated DNA was washed twice with 70% ethanol, dried, and dissolved in 200 μL of 10 mmol/L Tris/HCl, 0.1 mmol/L EDTA, 100 mmol/L NaCl (pH 7.0) for its enzymatic digestion, as previously described [71]. In brief, 5 μg DNA/μL (total DNA, 200 μg) was incubated with 100 U of DNase I in 40 μL Tris/HCl (10 mmol/L and 10 μL of 0.5 mol/L MgCl_2_ (final concentration of 20 mmol/L) at 37 °C for 1 h. The pH of the reaction mixture was then lowered with 15 μL of sodium acetate 0.5 mol/L to pH 5.1. Next, 10 μL of nuclease P1 (5 U) and 30 μL of 10 mmol/L ZnSO_4_ were added to give a final concentration of 1 mmol/L, and the mixture was incubated for 1 h. After readjusting the pH with 100 μL of 0.4 mol/L Tris/ClH (pH 7.8) followed by the addition of 20 μL alkaline phosphate (3 U), the samples were incubated for 30 min. Enzymes were precipitated with acetone (5 vol.), removed by centrifugation, and the supernatant evaporated to dryness.

DNA hydrolysates were dissolved in HPLC grade water and filtered through a 0.2-μm syringe filter before applying the samples to a Waters ODS HPLC column (2.5 × 0.46 ID; 5 μm particle size). The amount of 8-oxo-deoxiguanosine (8-oxo-dG) and deoxyguanosine (dG) in the DNA digest was measured by electrochemical and UV absorbance detection, under the elution conditions previously described [71]. Standard samples of dG and 8-oxo-dG were analyzed to ensure good separation and to allow for identification of those derived from cell DNA.

### 3.4. mRNA Extraction and Measurement

Total RNA was extracted from blood mononuclear cells purified by the Ficoll-Hypaque method and the Trizol method using a chloroform extraction protocol [75]. One microgram of total RNA was treated with DNase I (Roche, Mannheim, Germany) and was reverse-transcribed to cDNA using Ready-To-Go You Prime First-Strand beads (Amersham Pharmacia Biotech). Primers for PCR were designed by Primer3 program [76] (Table S1). The PCR reaction was done using SYBR Green PCR Master Mix and ABI PRISM 7000 Sequence Detection System (Applied Biosystems, Foster City, CA). To calculate the absolute number of copies, standard curves for each gene were plotted with a quantified cDNA template during each real-time PCR reaction and normalized to average mRNA values between glyceraldehyde-3-phosphate dehydrogenase (*GAPDH*) and beta 2 microglobulin (*B2M*) as previously described [77]. The mRNA values for each gene analyzed were expressed as the log ratio between interest gene mRNA value and the average value of housekeeping gene mRNA. Consequently, if the values of the mRNA levels are significantly higher in the groups of patients versus controls, it means that the gene is up-regulated. In contrast, if the values of mRNA levels are significantly lower in disease groups versus controls, it means that the gene is down-regulated. All the mRNA measurements of patients and controls were performed under the same conditions and at the same time.

### 3.5. Statistical Analysis

For each variable, the values were expressed as mean ± standard deviation or standard error values as indicated. The differences between each of the CV risk factors and the control group were calculated using Student t test for continuous variables. Proportions were compared with contingency tables and the χ^2^ test or the Fisher exact test (*n* > 5). Multivariate linear regression analyses were used to estimate the independent contributions of the age, gender and body mass index (BMI) to the mean baseline oxidative stress parameters, antioxidant enzyme activities and the relative mRNA levels of oxidant and antioxidant genes after log-transformation. Two-tailed values of *p* < 0.05 were considered as statistically significant.

## 4. Conclusions

According to the observed changes in mRNA and the OS level, it can be hypothesized that the GSH system plays a larger role than the TXN system does in hypertension. The TXN system is unable to control the increased ROS-generation when an inadequate response by the GSH system exists because OS is strongly enhanced. The different behavior of the two systems previously described in HT was also observed in FH and FCH, although the TXN system seems to be more stimulated in the presence of higher OS levels. Such is the case of HT.

Over the past few years, different reports have suggested that free radical production underlies the pathophysiological mechanism of atherosclerosis, proposing an antioxidant intervention to ameliorate or prevent it. Strategies focusing on combatting vascular disease through the inhibition of superoxide-generating enzymes or by increasing the intake of antioxidant substances have been proposed. The possibility that an impaired response of some of the antioxidant mechanisms exists offers a new approach for reducing OS. Although the clinical significance of this phenomenon is not well understood, it opens the door to a new therapeutic approach to reducing ROS by enhancing the antioxidant enzyme expression. Further studies are necessary to delineate the factors involved in the disturbance of the regulation of antioxidant enzymes and to point out areas of potential intervention.

## Supplementary Information



## Figures and Tables

**Table 1:** General characteristics of the study population.

Variables	Controls (*n* = 20)	FH (*n* = 17)	FCH (*n* = 30)	HT (*n* = 43)
Age (year)	39.9(14.3)	42.6(13.3)	45.6(8.5)	46.3(9.6)
Gender (M/F)	12/8	3/14	20/10 +	26/17 +
Body mass index (kg/m^2^)	25.9(3.0)	25.5(3.6)	26.9(3.9)	30.6(5.1) *,+,Ω
Office SBP (mmHg)	121.6(11.0)	120.2(10.7)	139.5(5.9) *,+	157.8(21.3) *,+, Ω
Office DBP (mmHg)	76.8(5.8)	73.3(9.1)	87.3(4.0) *,+	99.7(12.7) *,+, Ω
24-h SBP (mmHg)	-	-	-	142.0(16.9)
24-h DBP (mmHg)	-	-	-	90.5(10.5)
Baseline glucose (mg/dL)	89.7(6.4)	88.4(8.9)	101.3(16.4) *,+	104.3(20.4) *,+
Total-cholesterol (mg/dL)	189.7(35.9)	304.8(65.7) *	271.9(57.5) *	208.5(33.7) +, Ω
HDL-cholesterol (mg/dL)	47.6(9.7)	58.1(13.4) *	40.1(9.9) *,+	44.8(8.9) +, Ω.
Triglycerides (mg/dL)	102.2(48.6)	127.5(51.9)	294.4(175.1) *,+	146.2(71.6) Ω

All values are indicated as mean (standard deviation). All differences are significant after adjusting for age, gender and Body Mass Index;

* *p* values denote differences between controls and disease;

+ *p* values denote differences between FH and others diseases;

Ω *p* values denote differences between FCH and HT.

**Table 2:** Oxidative stress, byproducts and antioxidant enzymes activity in the study.

Variables	Controls (*n* = 20)	FH (*n* = 17)	FCH (*n* = 30)	HT (*n* = 43)
GSH	22.9 ± 0.9	18.3 ± 1.1 *	18.7 ± 0.6 *	15.7 ± 0.6 *, Ω
GSSG	0.28 ± 0.04	0.43 ± 0.04 *	0.32 ± 0.02 +	1.13 ± 0.05 *,+, Ω
GSSG/GSH	1.3 ± 0.2	2.5 ± 0.3 *	1.8 ± 0.2 *	7.8 ± 0.4 +, Ω
MDA	0.23 ± 0.02	0.30 ± 0.04	0.26 ± 0.02	0.92 ± 0.29 *,+, Ω
8-oxo-dG	4.8 ± 0.3	5.5 ± 0.3	5.7 ± 0.2 *	6.8 ± 0.2 *,+, Ω
Catalase	217.0 ± 10.1	140.2 ± 11.4 *	173.3 ± 10.3 *	110.9 ± 4.5 *,+, Ω
GPX1	58.5 ± 1.8	54.1 ± 2.1	51.7 ± 1.2 *	32.8 ± 0.9 *,+, Ω
SOD	6.9 ± 0.6	5.1 ± 0.7	5.6 ± 0.7	3.8 ± 0.3 *,+, Ω

All values denote mean ± standard error. All differences are significant after adjusting for age, gender and Body Mass Index. GSH: reduced glutathione (μmol/mg protein); GSSG: oxidized glutathione (μmol/mg protein); MDA: malondialdehide (μmol/mg protein); 8-oxo-dG: 8-oxo-2′-deoxyguanosine. The value of 8-oxo-dG was expressed as the number of oxidized bases/106 deoxyguanosine. Catalase, GPX1 and CuZn-SOD activities were expressed as U/protein

* *p* values denote differences between controls and disease.

+ *p* values denote differences between FH and others disease.

Ω *p* values denote differences between FCH and HT. NOTE: see Table 1 for comparison.
