# Supplementary material for: Different Impacts of Cardiovascular Risk Factors on Oxidative Stress
Source: Int J Mol Sci. 2011 Sep 20;12(9):6146–63. doi: 10.3390/ijms12096146 (PMC3189774; doi:10.3390/ijms12096146)
Supplement: Supplementary file 1 [file ijms-12-06146-s001.pdf]

Supplementary Information

Maria L. Mansego [1,2,*], Josep Redon [2,3], Sergio Martinez-Hervas [4,5], Jose T. Real [4,5], Fernando Martinez [2,3], Sebastian Blesa [1], Veronica Gonzalez-Albert [1], Guillermo T. Saez [6], Rafael Carmena [4,5] and Felipe J. Chaves [1,5]

[1] Genotyping and Genetic Diagnosis Unit, Research Foundation of Hospital Clínico. Av. Blasco Ibañez, 17, Valencia 46010, Spain; E-Mails: Sebastian.Blesa@uv.es (S.B); veronica.gonzalez@uv.es (V.G.-A.); felipe.chaves@uv.es (F.J.C.)
[2] CIBER of obesity (CIBERob), Santiago de Compostela 15706, Spain; E-Mails: Josep.redon@uv.es (J.R.); fernandoctor@hotmail.com (F.M.)
[3] Hypertension Unit, Hospital Clinico. Av. Blasco Ibañez, 17, Valencia 46010, Spain
[4] Service of Endocrinology and Nutrition, Hospital Clínico Universitario Av. Blasco Ibañez, 17, Valencia 46010, Spain; E-Mails: Sergio.Martinez@uv.es (S.M.-H.); Jose.T.Real@uv.es (J.T.R.); Rafael.Carmena@uv.es (R.C.)
[5] CIBER de Diabetes y Enfermedades Metabólicas Asociadas (CIBERDEM), Barcelona 08017, Spain
[6] Department of Biochemistry and Molecular Biology, University of Valencia. Av. Blasco Ibañez, 17, Valencia 46010, Spain; E-Mail: Guillermo.Saez@uv.es

* Author to whom correspondence should be addressed; E-Mail: m.luisa.mansego@uv.es; Tel.: +34-963-983-916; Fax: +34-963-864-926.

Received: 10 June 2011; in revised form: 1 September 2011 / Accepted: 7 September 2011 / Published: 20 September 2011

Abstract: The objective of the study was to evaluate oxidative stress (OS) status in subjects with different cardiovascular risk factors. With this in mind, we have studied three models of high cardiovascular risk: hypertension (HT) with and without metabolic syndrome, familial hypercholesterolemia (FH) and familial combined hyperlipidemia (FCH) with and without insulin resistance. Oxidative stress markers (oxidized/reduced glutathione ratio, 8-oxo-deoxyguanosine and malondialdehide) together with the activity of antioxidant enzyme triad (superoxide dismutase, catalase, glutathione peroxidase) and activation of both pro-oxidant enzyme (NAPDH oxidase components) and AGTR1 genes, as well as antioxidant enzyme genes (CuZn-SOD, CAT, GPX1, GSR, GSS and TXN) were measured in mononuclear cells of controls (n = 20) and patients (n = 90) by assessing mRNA levels. Activity of some of these antioxidant enzymes was also tested. An increase in OS and pro-oxidant gene mRNA values was observed in patients compared to controls. The hypertensive group showed not only the highest OS values, but also the highest pro-oxidant activation compared to those observed in the other groups. In addition, in HT a significantly reduced antioxidant activity and mRNA induction of antioxidant genes were found when compared to controls and the other groups. In FH and FCH, the activation of pro-oxidant enzymes was also higher and antioxidant ones lower than in the control group,

although it did not reach the values obtained in hypertensives. The thioredoxin system was more activated in patients as compared to controls, and the highest levels were in hypertensives. The increased oxidative status in the presence of cardiovascular risk factors is a consequence of both the activation of pro-oxidant mechanisms and the reduction of the antioxidant ones. The altered response of the main cytoplasmic antioxidant systems largely contributes to OS despite the apparent attempt of the thioredoxin system to control it.

Keywords: oxidative stress; glutathione peroxidase; superoxide dismutases; mRNA; hypertension; familial hypercholesterolemia; combined familial dyslipidemia

---

Table S1. Oligonucleotides used in real-time quantitative RT-PCR.

Gene	NCBI ID	Forward Sequence	Reverse Sequence
B2M	NM_004048	CCCCAAATTCTAAGCAGAGTATGTAA	TCTTCAATCTCTTGCACTCAAAGC
GAPDH	NM_002046	GAAGGTGAAGGTCGGAGTCAAC	CTGGAAGATGGTGATGGGATTTC
AGTR1	NM_000685	GAATACCGCTGGCCCTTTG	AGGTATCGATCAATGCTGAGACAC
P22PHOX	NM_000101	CCGGCCTGATCCTCATCAC	ACGGCGGTCATGTACTTCTGTC
P91PHOX	NM_000397	AACTGCATGCTGATTCTCTTGC	ATAGATGTGCAATGGTGTGAATCG
P47PHOX	NM_000265	CGTACCCAGCCAGCACTATGTGTA	AACCACTTGGGAGCTGGGAGGT
P67PHOX	NM_000433	CGAGGGATGCTCTACTACCAGAC	TATAACACCTCACAGGCAAACAGC
RAC1	NM_006908	GGTGGGAGACGGAGCTGTAGGTA	GGGGCGTAATCTGTCATAATCTTC
SOD1	NM_000454	GGTGTGGCCGATGTGTCTATT	CCAGCGTTTCCTGTCTTTGTACTT
SOD2	NM_001024465	GTGGAGAACCCAAAGGGGAGTT	GTGGAATAAGGCCTGTTGTTCCTT
SOD3	NM_003102	GCGGAGCCCAACTCTGACTC	CTGCATGACCTCCTGCCAGAT
GSR	NM_000637	ATCCCCGGTGCCAGCTTAGG	AGCAATGTAACCTGCACCAACAA
GSS	NM_000178	ACTCACTGGGATGTGGGTGAAGAAG	TCCTCCCCATATAGGTTGTTACCTC
CAT	NM_001752	GTTACTCAGGTGCGGGCATTCTAT	GAAGTTCTTGACCGCTTTCTTCTG
GPX1	NM_201397	AACCAGTTTGGGCATCAGGAGAAC	ATGAGCTTGGGGTCGGTCATAAG
GPX4	NM_001039847	CTGCTCTGTGGGGCTCTGG	ACGAAGCCCCGGTACTTGTC
TXN	NM_003329	AGACTCCAGCAGCCAAGATGG	GAGAGGGAATGAAAGAAAGGCTTG
TXN2	NM_012473	CACTTCCAGAGCCCTGCAGAC	CACTGGTGTCTCACTGTTGACCAC

Table S2. General characteristics and oxidative parameters of hypertensive population grouped by metabolic syndrome status and familial combined hyperlipidemia with and without insulin resistance.

Variables	Non-MS(n = 21)	MS(n = 22)	Non-IR(n = 13)	IR(n = 17)
Age (yr)	43.5 (9.1)	49.3 (9.1)	45.8 (8.3)	45.5 (8.9)
Gender (M/F)	12/9	15/7	8/5	12/5
Waist (cm)	--	--	87.2 (15.6)	96.6 (11.8)
Body mass index (kg/m2)	29.0 (5.5)	32.2 (4.1)*	26.2 (3.3)	27.4 (4.3)
Office SBP (mmHg)	154.0 (15.2)	162.2 (26.4)	140.0 (5.9)	139.3 (6.7)
Office DBP (mmHg)	97.0 (11.6)	102.7 (13.5)	88.5 (4.0)	86.8 (4.7)
24-hour SBP (mmHg)	138.6 (14.0)	145.7 (19.3)	--	--
24-hour DBP (mmHg)	89.1 (10.2)	92.1 (10.8)	--	--
Baseline glucose (mg/dL)	99.8 (7.2)	109.6 (28.7)	99.3 (19.5)	102.8 (14.1)
Total-cholesterol (mg/dL)	204.0 (29.5)	213.4 (38.0)	294.5 (48.3)	254.5 (59.1)
HDL-cholesterol (mg/dL)	47.9 (8.4)	41.6 (8.5) *	43.9 (11.3)	37.2 (7.9)
Triglycerides (mg/dL)	105.0 (44.7)	189.3 (69.3) *	291.0 (215.7)	297.0 (143.8)
GSH	15.1 (3.3)	15.4 (3.8)	19.3 (3.0)	18.1 (3.3)
GSSG	1.10 (0.36)	1.21 (0.39)	0.28 (0.09)	0.36 (0.10)*
GSSG/GSH	7.7 (3.2)	8.4 (3.5)	1.5 (0.5)	2.0 (0.7)*
MDA	1.09 (1.72)	0.76 (0.14)	0.28 (0.13)	0.25 (0.08)
8-oxo-dG	6.7 (1.1)	6.8 (0.8)	5.6 (0.9)	5.9 (0.9)
Catalase	107.9 (11.4)	110.1 (9.8)	170.4 (50.5)	169.3 (59.3)
GPX1	32.2 (4.0)	33.4 (4.3)	50.6 (5.2)	52.3 (5.4)
SOD	3.6 (0.6)	4.0 (1.0)	5.0 (3.2)	6.2 (4.4)

Values are mean (standard deviation) Non-MS: HT without metabolic syndrome; MS: HT with metabolic syndrome; Non-IR: familial combined hyperlipidemia without insulin resistance; IR: familial combined hyperlipidemia with insulin resistance. GSH: reduced glutathione (µmol/mg protein); GSSG: oxidized glutathione (µmol /mg protein); MDA: malondialdehide (µmol/mg protein); 8-oxo-dG: 8-oxo-2'-deoxyguanosine. The value of 8-oxo-dG was expressed as the number of oxidized bases/10^6 deoxyguanosine. Catalase, GPX1 and CuZn-SOD activities were expressed as U/protein. *: *p* values denote differences between Non-MS and MS or Non-IR and IR.

© 2011 by the authors; licensee MDPI, Basel, Switzerland. This article is an open access article distributed under the terms and conditions of the Creative Commons Attribution license (http://creativecommons.org/licenses/by/3.0/).
